# Variation in the Management of Test Results after Hospital Discharge: A Pediatric Safety Concern

**DOI:** 10.1097/pq9.0000000000000871

**Published:** 2026-02-23

**Authors:** Meenu Krishnasamy, Rhiya Sharma, Lydia Nashed, Khoa Luong, Courtney Port

**Affiliations:** From the * Department of Pediatrics, Inova Children’s Hospital, Falls Church, Va.; † Department of Pediatrics, University of Chicago, Chicago, Ill.; ‡ University of Virginia School of Medicine, Falls Church, Va.

## Abstract

**Introduction:**

Delay or failure to follow up tests pending at discharge (TPAD) is a major safety concern and a focus in the literature, but few studies exist in the pediatric context. We aimed to explore pediatric hospital medicine physicians’ practices and perceptions of the management of TPAD.

**Methods:**

Surveys were provided to hospitalists and residents at a single institution using selected questions from a published national adult primary care survey and newly developed questions. Results were analyzed using descriptive statistics and Fisher’s exact tests.

**Results:**

Surveys were completed by 25 hospitalists (89%) and 25 residents (68%). Forty-four percent (n = 11) of hospitalists reported missing test results, yet only 18% (n = 2) agreed that this error led to a delay in care. Seventy-six percent (n = 19) of hospitalists reported lacking adequate assistance to manage TPAD. There is uncertainty about who is responsible for managing TPAD among 88% (n = 22) of hospitalists and 92% (n = 23) of residents. Hospitalists were more likely than residents to use cognitive reminders (72% versus 20%, *P* = 0.0005). Many hospitalists (80%, n = 20) and residents (80%, n = 20) agreed that TPAD management is a safety issue, and nearly all hospitalists (88%, n = 22) and residents (96%, n = 24) want a standardized process. Residents were more likely to desire a shared responsibility (68% versus 32%, *P* = 0.02). Only 56% (n = 14) of hospitalists had formal education in result management; 76% (n = 19) of residents desired training.

**Conclusions:**

Pediatric hospitalists and residents are concerned about TPAD management, and there is considerable variability in current practices. Developing a standard process is supported by nearly all.

## INTRODUCTION

### Index Case

Overnight, a pediatric patient with an expected discharge for the following day reported dysuria, prompting the resident physician to order a urine culture. Due to miscommunication, the discharging team was unaware of the pending result. The urine culture, which showed more than 100,000 colony forming units/mL *Escherichia coli*, was not followed up after discharge. Several days later, the patient returned with urosepsis, requiring intensive care unit–level care before making a full recovery. A brief exploration of test result management after discharge revealed that the average time to mark an in-basket result as “done” during the past year was 8.4 days among all inpatient providers. Notably, there was significant variation across departments, ranging from 0.4 to 44 days.

### Background

Within the inpatient medical system, the follow-up of tests pending at discharge (TPAD) is an ongoing patient safety concern identified by several safety organizations.^1,2^ About 40% of patients leave the hospital with at least 1 TPAD.^3^ In 1 systematic review, there was a lack of follow-up on 20%–61% of test results from discharged hospitalized patients; this issue was particularly significant for critical test results.^4^ Although laboratories have policies for critical test result notification, there is great heterogeneity in the content of critical test alert lists.^5^ Depending on patient characteristics, even results of tests deemed “noncritical” could be clinically significant, as demonstrated by our index case. Failure to follow up on TPAD increases the risk of missed or delayed diagnoses, leading to suboptimal clinical outcomes. It may have serious medicolegal implications.^6^ Following the 21st Century Cures Act,^7^ many institutions now release results directly to patient portals. Delays or failures by providers to address these results with patients can erode trust in the healthcare system.^8,9^

Many physicians are aware of this gap in patient safety. Eighty-three percent of surveyed general adult practitioners reported encountering at least 1 delayed test result that affected patient care during the last 2 months.^10^ Additionally, about 30% of primary care providers (PCPs) surveyed reported seeing at least 1 patient in the last month with a missed test result from their inpatient admission.^10^ However, few studies exist describing TPAD management in the pediatric context.^11,12^ Studies among adult patients^13–16^ note large variability in addressing TPAD, a lack of communication between the discharging team and PCP, ambiguity of responsibility, and delays in addressing actionable results. Our study aimed to explore TPAD management practices and perceptions among pediatric hospital medicine (PHM) clinicians at a large suburban center.

## METHODS

We conducted a cross-sectional, electronic survey of clinicians at a large mid-Atlantic children’s hospital with 226 pediatric beds and 7000 pediatric admissions annually. Our institution uses Epic Systems Corporation’s electronic health record (Epic, Verona, WI), one of the most widely used systems globally.^17^

The study population included attending PHM physicians (hospitalists), PHM fellows, and pediatric resident physicians. We recruited participants via email and at department meetings. Our team conducted a local needs assessment from July 2023 to October 2023, and our local institutional review board approved the study.

### Data Collection

We used a 23-item electronic survey developed through literature review, iterative content review, and pilot testing to collect data. (**See Supplemental Digital Content 1**, which displays survey questions, https://links.lww.com/PQ9/A740.) We selected content areas and 14 survey questions from a previously published national survey.^18^ Focus areas included hardware and software content, user interface, workflow and communication, organizational features, and responder characteristics. We created additional questions to assess perceived time spent, attitudes about TPAD as a safety issue, and prior training on result management. Because residents do not independently review TPAD results at our institution, we selected 13 questions from the hospitalist survey for a shortened resident survey. The survey questions were multiple choice, with most items scored on a 5-point Likert scale or a yes/no/unsure scale.

We administered the survey electronically and anonymously via REDCap, a Health Insurance Portability and Accountability Act–compliant software platform.^19,20^ The survey was beta-tested by the authors, pediatric chief residents, and PHM advanced practice providers for readability and clarity.

### Outcomes and Analysis

We generated descriptive statistics to summarize responder characteristics and aggregate responses. We used Fisher’s exact test, with *P* values less than 0.05 considered significant, to determine differences between groups. This study was approved by our local review board (INOVA-2024-61).

## RESULTS

A total of 50 physicians completed the survey: 23 hospitalists (81% response rate), 2 PHM fellows (100% response rate), and 25 resident physicians (68% response rate). We grouped the fellows with hospitalists to preserve anonymity. Forty-four percent (n = 11) of hospitalists reported more than 10 years since residency/fellowship, 32% (n = 8) reported 6–10 years, and 24% (n = 6) reported 1–5 years. (**See table 1, Supplemental Digital Content 2**, which demonstrates responder demographics and current practices, https://links.lww.com/PQ9/A741.) Of the 25 resident physicians, 7 (28%) were PGY-1, 11 (44%) were PGY-2, and 7 (28%) were PGY-3.

**Fig. 1.**
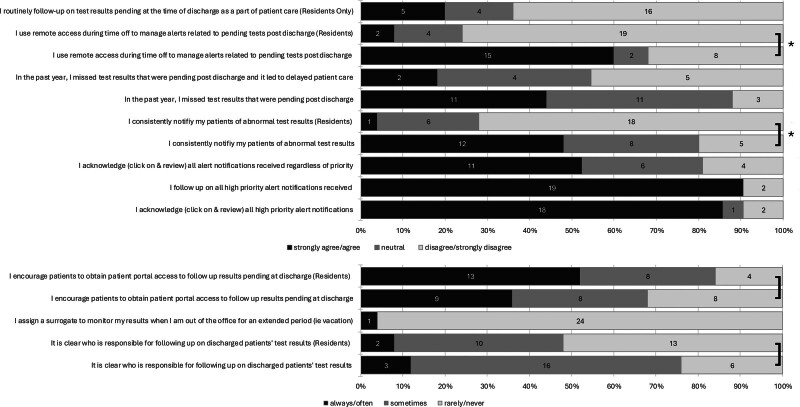
Provider practices. *P < 0.001.

Forty-four percent (n = 11) of hospitalists reported missing results in the past year, whereas another 44% (n = 11) were unsure. Despite this, only 18% (n = 2) of those reporting missed results agreed that it led to a delay in patient care, whereas 36% (n = 4) were neutral and 46% (n = 5) disagreed (Fig. 1). Hospitalists who reported missing a test result in the past year, compared with those who did not or were unsure, were more likely to agree that there are too many alerts to easily focus on the most important ones (100%, n = 11, versus 57%, n = 8; *P* = 0.02) (**Supplemental Digital Content 2**, https://links.lww.com/PQ9/A741).

Seventy-six percent (n = 19) of hospitalists and 60% (n = 15) of residents reported lacking the support needed to manage TPAD, and 72% (n = 18) of hospitalists and 20% (n = 5) of residents use manual cognitive reminders to follow up on results (Table 1). Of those hospitalists and residents who use at least 1 cognitive reminder (n = 23, 46%), the most frequently used cognitive reminder was manually putting the patient on a personal follow-up list within the electronic medical record (EMR) (65%, n = 15), followed by using a personal reminder in the EMR for notification of the result (26%, n = 6)—4 hospitalists (17%) used notes/to-do lists outside of the EMR as cognitive reminders.

**Table 1.** Responder Use of Result Management Information Technology Tools

Use of Information Technology Tools	Yes		No		Unsure		*P* (Yes Versus No/Unsure)
I use the in-basket to follow up on TPAD	21	84%	4	16%	n/a	n/a	
I use the sort function in my in-basket results according to urgency, patient name, location, or alert date/time	2	8%	18	72%	1	4%	
I have remote access to the EMR	23	92%	2	8%	n/a	n/a	0.99
Residents	24	96%	0	0%	1	4%	
I use the mobile EMR App on my personal cell phone	17	68%	8	32%	n/a	n/a	**0.004**
Residents	24	100%	0	0%	0	0%	
I use at least 1 manual cognitive reminder for TPAD	18	72%	7	28%	n/a	n/a	**0.0005**
Residents	5	20%	20	80%	n/a	n/a	
Cognitive reminder: add to a reminder list in the EMR	4	16%	21	84%	n/a	n/a	0.67
Residents	2	8%	23	92%	n/a	n/a	
Cognitive reminder: add to a personal follow-up list in the EMR	11	44%	14	56%	n/a	n/a	0.06
Residents	4	16%	21	84%	n/a	n/a	
Cognitive reminder: personal electronic or handwritten notes/to-do list	4	16%	21	84%	n/a	n/a	0.11
Residents	0	0%	25	100%	n/a	n/a	
Cognitive reminder: none of the above, I only use the in-basket results	7	28%	18	72%	n/a	n/a	**0.0005**
Residents	20	80%	5	20%	n/a	n/a	
Cognitive reminder: other	0	0%	25	100%	n/a	n/a	0.99
Residents	1	4%	24	96%	n/a	n/a	
I use the patient portal to notify patients of test results	1	4%	24	96%	0	0%	0.99
Residents	2	8%	20	80%	3	12%	

All responses are from hospitalists, unless otherwise stated. The statistical test used to compare hospitalists and residents were Fisher’s exact test. Bold indicates statistical significance at *P* < 0.05. Not all respondents answered all questions.

n/a, not applicable.

There is ambiguity about who is responsible for managing TPAD, with only 12% (n = 3) of hospitalists and 8% (n = 2) of residents reporting that it is always or often clear who is responsible for following up on TPAD. Forty-eight percent (n = 12) of hospitalists agreed that they consistently notify patients or PCPs of abnormal results, whereas 32% (n = 8) neither agreed nor disagreed, and 20% (n = 5) disagreed or strongly disagreed. Among residents, 72% (n = 18) disagreed that they consistently notify patients or PCPs of abnormal results, whereas only 4% (n = 1) agreed. These differences between hospitalist and resident responses were statistically significant (*P* < 0.001) (Fig. 1; **Supplemental Digital Content 2**, https://links.lww.com/PQ9/A741).

**Fig. 2.**
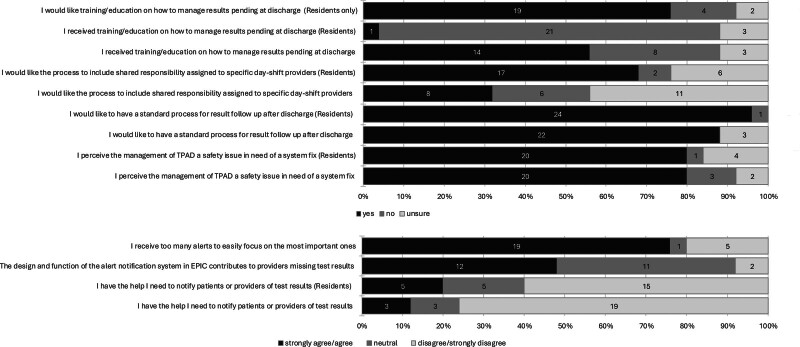
Attitudes, perceptions, and preferences.

Eighty percent (n = 20) of hospitalists and 80% (n = 20) of residents agreed or strongly agreed that the management of TPAD is a safety issue requiring a system solution. Eighty-eight percent (n = 22) of hospitalists and 96% (n = 24) of residents agreed with creating a standardized process for TPAD management. Additionally, 56% (n = 14) of hospitalists and 4% (n = 1) of residents had formal education in result management. Seventy-six percent (n = 19) of residents desire training (Fig. 2). (**See table 2, Supplemental Digital Content 3**, which demonstrates responder attitudes, perceptions, and preferences, https://links.lww.com/PQ9/A742.)

## DISCUSSION

This study of pediatric hospitalists and residents described current practices, the use of information technology tools, and attitudes and preferences regarding the management of TPAD. Overall, there is a widespread acknowledgement of TPAD as a patient safety issue. Similar to studies in the adult literature,^15,16^ our study found high levels of uncertainty regarding responsibility for managing TPAD, with only 12% of hospitalists and 8% of residents reporting that there is always or often clarity on who should follow up on results. Given such uncertainty, the variability among hospitalists we found in PCP and caregiver notification of abnormal test results is no surprise. Differences in notification between residents and hospitalists are likely due to residents’ limited experience with TPAD management. Variability in hospitalist notification practices aligns with prior studies showing vulnerability at transition points in care, such as hospital^21–24^ and emergency room discharge.^25^ This is especially true for health systems such as ours, where PCPs and specialists may not use the same EMR as the hospital.^10^

Many (72%) hospitalists reported relying on manual cognitive reminders, which may suggest that existing EMR tools are inadequate to support physicians in this process. Suboptimal use or design issues in the EMR can cause providers to miss results.^21–24^ High cognitive workloads can also lead to avoidance or cognitive dismissal of results.^26^ This is consistent with our study, where those who reported missing results, compared with those who did not or were unsure, were more likely to cite alert fatigue as a problem (100% versus 57%). These findings underscore the need for a process to reduce cognitive burden during TPAD management. Overall, both hospitalists (80%) and residents (80%) agreed that inpatient physicians require a system-based solution to manage TPAD.

Only 18% of hospitalists reported missing results during the past year, leading to care delays, compared with 83% of adult outpatient physicians who reported patient care delays during the past 2 months.^10^ This difference may stem from limited continuity of care, leading hospitalists to be unaware of delays in care. Limitations of our study include recall bias and other inaccuracies in self-reported data, as well as its single-institution design, which limits the generalizability of the results. The lower response rate by residents and the overall small number of providers limited our ability to analyze practices and perceptions by provider characteristics. Further research is needed to determine if results are similar across different PHM contexts.

**Fig. 3.**
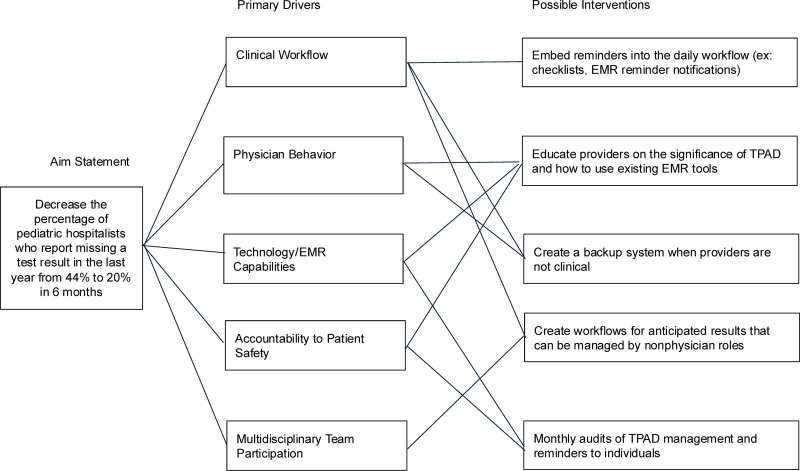
Driver diagram.

To improve the safety of test result management within departments, we suggest using a quality improvement methodology with a SMART (Specific, Measurable, Achievable, Realistic, Time-based) aim to decrease the percentage of pediatric hospitalists who report missing a test result in the last year from 44% to 20% in 6 months (Fig. 3). Additional quality improvement measures of interest could include the frequency of diagnostic delays (outcome), turnaround time for result management (process), and provider satisfaction (balancing). Interventions should focus on reducing variability by clarifying responsibilities and establishing a standard workflow. Provider education and feedback have been shown to improve rates of result management.^27^ This education should focus on optimizing or creating new EMR result management tools, determining the urgency of a result, and expectations and methods for patient and other care team provider notification and documentation. Practices, especially those doing shift work, should also consider developing a process with shared responsibility, using interprofessional teams^28^ for postdischarge result management.

## CONCLUSIONS

Pediatric hospitalists and residents are concerned about missing results after discharge and agree that a system-based solution is needed. We found considerable variability in current practices and a consistent desire for a standard TPAD management process. Next steps include a quality initiative and the development of a trainee curriculum to improve TPAD management.

## Supplementary Material

## References

[1] World Alliance for Patient Safety. Summary of the evidence on patient safety: implications for research. 2008. World Health Organization. Available at https://iris.who.int/server/api/core/bitstreams/10c19ae7-2759-49b0-a6e8-b8f30546d682/content. Accessed June 15, 2025.

[2] ECRI Institute. Top 10 patient safety concerns for healthcare organizations. 2017. Available at https://www.ecri.org/emailresources/psrq/top10/2017_pstop10_executivebrief.pdf. Accessed March 15, 2025.

[3] Were MC Li X Kesterson J. Adequacy of hospital discharge summaries in documenting tests with pending results and outpatient follow-up providers. J Gen Intern Med. 2009;24:1002–1006. 19575268 10.1007/s11606-009-1057-y PMC2726888

[4] Callen J Georgiou A Li J. The safety implications of missed test results for hospitalised patients: a systematic review. BMJ Qual Saf. 2011;20:194–199. 10.1136/bmjqs.2010.044339 PMC3038104 21300992

[5] Truijens K Frans G Vermeersch P. Critical results in laboratory medicine. Clin Chem. 2024;70:1220–1230. 39245958 10.1093/clinchem/hvae120

[6] Graber ML Franklin N Gordon R. Diagnostic error in internal medicine. Arch Intern Med. 2005;165:1493–1499. 16009864 10.1001/archinte.165.13.1493

[7] Office of the National Coordinator for Health Information Technology, US Department of Health and Human Services. 21st century cures act: interoperability, information blocking, and the ONC health it certification program. Fed Regist. 2020;85:25642–25961.

[8] Steitz BD Turer RW Lin C. Perspectives of patients about immediate access to test results through an online patient portal. JAMA Netw Open. 2023;6:e233572. 36939703 10.1001/jamanetworkopen.2023.3572 PMC10028486

[9] Giardina TD Baldwin J Nystrom DT. Patient perceptions of receiving test results via online portals: a mixed-methods study. J Am Med Inform Assoc. 2018;25:440–446. 29240899 10.1093/jamia/ocx140 PMC5885801

[10] Poon EG Gandhi TK Sequist TD. “I wish I had seen this test result earlier!”: dissatisfaction with test result management systems in primary care. Arch Intern Med. 2004;164:2223–2228. 15534158 10.1001/archinte.164.20.2223

[11] Ferris TG Johnson SA Co JPT. Electronic results management in pediatric ambulatory care: qualitative assessment. Pediatrics. 2009;123(suppl_2):S85–S91. 19088235 10.1542/peds.2008-1755G

[12] Shriner AR Baker RM Ellis A. Improving follow-up of tests pending at discharge. Hosp Pediatr. 2021;11:1363–1369. 34849927 10.1542/hpeds.2021-006000

[13] Bodley T Kwan JL Matelski J. Test result management practices of Canadian internal medicine physicians and trainees. J Gen Intern Med. 2019;34:118–124. 30298242 10.1007/s11606-018-4656-7 PMC6318178

[14] Wahls T Haugen T Cram P. The continuing problem of missed test results in an integrated health system with an advanced electronic medical record. Jt Comm J Qual Patient Saf. 2007;33:485–492. 17724945 10.1016/s1553-7250(07)33052-3

[15] Hysong SJ Sawhney MK Wilson L. Understanding the management of electronic test result notifications in the outpatient setting. BMC Med Inform Decis Mak. 2011;11:22. 21486478 10.1186/1472-6947-11-22 PMC3100236

[16] Menon S Murphy DR Singh H. Workarounds and test results follow-up in electronic health record-based primary care. Appl Clin Inform. 2016;7:543–559. 27437060 10.4338/ACI-2015-10-RA-0135 PMC4941859

[17] Chishtie J Sapiro N Wiebe N. Use of Epic electronic health record system for health care research: scoping review. J Med Internet Res. 2023;25:e51003. 38100185 10.2196/51003 PMC10757236

[18] Singh H Spitzmueller C Petersen NJ. Primary care practitioners’ views on test result management in EHR-enabled health systems: a national survey. J Am Med Inform Assoc. 2013;20:727–735. 23268489 10.1136/amiajnl-2012-001267 PMC3721157

[19] Harris PA Taylor R Thielke R. Research electronic data capture (REDCap)—a metadata-driven methodology and workflow process for providing translational research informatics support. J Biomed Inform. 2009;42:377–381. 18929686 10.1016/j.jbi.2008.08.010 PMC2700030

[20] Harris PA Taylor R Minor BL; REDCap Consortium. The REDCap consortium: building an international community of software partners. J Biomed Inform. 2019;95:1. 10.1016/j.jbi.2019.103208 PMC7254481 31078660

[21] Whitehead NS Williams L Meleth S. Interventions to improve follow-up of laboratory test results pending at discharge: a systematic review. J Hosp Med. 2018;13:631–636. 29489926 10.12788/jhm.2944 PMC9491200

[22] Dalal AK Roy CL Poon EG. Impact of an automated email notification system for results of tests pending at discharge: a cluster-randomized controlled trial. J Am Med Inform Assoc. 2014;21:473–480. 24154834 10.1136/amiajnl-2013-002030 PMC3994865

[23] Darragh PJ Bodley T Orchanian-Cheff A. A systematic review of interventions to follow-up test results pending at discharge. J Gen Intern Med. 2018;33:750–758. 29352419 10.1007/s11606-017-4290-9 PMC5910344

[24] El-Kareh R Roy C Williams DH. Impact of automated alerts on follow-up of post-discharge microbiology results: a cluster randomized controlled trial. J Gen Intern Med. 2012;27:1243–1250. 22278302 10.1007/s11606-012-1986-8 PMC3445692

[25] Li J Paoloni R Li L. Does health information technology improve acknowledgement of radiology results for discharged emergency department patients? A before and after study. BMC Med Inform Decis Mak. 2020;20:100. 32493463 10.1186/s12911-020-01135-9 PMC7268495

[26] Thomas J Dahm MR Li J. Variation in electronic test results management and its implications for patient safety: a multisite investigation. J Am Med Inform Assoc. 2020;27:1214–1224. 32719839 10.1093/jamia/ocaa093 PMC7481032

[27] Bayes LY Kronenfeld R Chang HT. Improving pediatric hospital medicine management of test results pending at discharge: a quality improvement initiative. Pediatrics. 2022;149:160–160.

[28] Vepraskas SH Chou E Hahn D. An interprofessional initiative to address tests pending at discharge for hospitalized pediatric patients. WMJ. 2024;123:29–33. 38436636

