# Supplementary material for: Variation in the Management of Test Results after Hospital Discharge: A Pediatric Safety Concern
Source: Pediatr Qual Saf. 2026 Feb 23;11(1):e871. doi: 10.1097/pq9.0000000000000871 (PMC12928903; doi:10.1097/pq9.0000000000000871)
Supplement: Supplementary file 1 [file pqs-11-e871-s001.pdf]

Supplement 1: Survey Questions

Practices and Perceptions on Management of Test Results Pending at Discharge

Pediatric Hospital Medicine Attending Physician and Fellow Survey

Current practices

In-basket Use

1) I acknowledge (ie, click on & review) all high priority alert notifications.

   strongly agree, agree, neutral, disagree, strongly disagree

2) I follow up on all high priority alert notifications received.

   strongly agree, agree, neutral, disagree, strongly disagree

3) I acknowledge (ie, click on & review) all alert notifications received regardless of priority.

   strongly agree, agree, neutral, disagree, strongly disagree

4) In the past year, I missed test results.

   strongly agree, agree, neutral, disagree, strongly disagree

5) In the past year, I missed test results that led to delayed patient care.

   strongly agree, agree, neutral, disagree, strongly disagree

6) I use remote access during time off to manage test results for discharged patients

   strongly agree, agree, neutral, disagree, strongly disagree

Patient/Provider Notification:

7) It is clear who is responsible for following up on a discharged patient's test results.

   always, often, sometimes, rarely, never

8) I assign a surrogate to take care of my In-Basket results when I am out of the office for an extended period (ie on vacation)

   always, often, sometimes, rarely, never

9) I consistently notify patients or PCP of abnormal test results

   strongly agree, agree, neutral, disagree, strongly disagree

10) I have the help needed for notifying patients of test results

   strongly agree, agree, neutral, disagree, strongly disagree

<u>Use of IT/Tools</u>:

11) I use my in-basket to follow up on discharged patients' test results.

   Yes, no, unsure

12) I use the sort function in my in-basket results according to urgency, patient name, location, or alert date/time.

   Yes, no, unsure

13) I have remote access to the EMR (ie citrix).

   Yes, no, unsure

14) I have the EMR mobile app (ie Haiku) on my personal cell phone.

   Yes, no, unsure

15) I use manual cognitive reminders to keep track of results pending at discharge (select all that apply):
    a) *Add the patient to a reminder list (with personal notification) within the EMR*
    b) *Add the patient to a personal lab follow-up list in the EMR*
    c) *Personal electronic or handwritten notes/to-do list outside of the EMR*
    d) *Other: (free text response)*
    e) *None of the above, I only use the in-basket results*

16) I use the patient portal (ie MyChart) to communicate test results to patients/families.

   Yes, no, unsure

17) I encourage patients/families to obtain access to the patient portal (ie MyChart) to see results for tests that are pending at discharge.

always, often, sometimes, rarely, never

18) I would like to receive feedback about my performance related to follow-up of high priority result notifications

    Yes, no, unsure

Attitude about IT (Information Technology) Solutions:

19) The alert notification system in my EMR makes it possible for providers to miss test results.

    strongly agree, agree, neutral, disagree, strongly disagree

20) I receive too many alerts to easily focus on the most important ones.

    strongly agree, agree, neutral, disagree, strongly disagree

21) I perceive pending tests at discharge a safety issue in need of a system fix.

    Yes, no, unsure

22) I would like our division to have a standard process for result follow-up after discharge.

    Yes, no, unsure

23) I would like that standard process to include shared responsibility assigned to specific day-shift providers.

    Yes, no, unsure

Responder Characteristics (Demographic Data)

- Number of years out of residency

  1 to 5 years, 6 to 10 years, 11 to 15 years, >15 years

- Number of years you have used an EMR

  1 to 5 years, 6 to 10 years, 11 to 15 years, >15 years

- Following up on results after patient discharge was part of my training on hospital medicine rotations during residency/fellowship.

  Yes, no, unsure

Practices and Perceptions on Management of Test Results Pending at Discharge

Resident Survey

Please help us gather information for a needs assessment on resident perceptions & practices on the management of results on tests that are pending at the time of hospital discharge. When answering these questions, consider only your hospital rotations (wards/PHM, PICU, NICU, ED). Thank you!

Current practice

<u>In-basket Use:</u>

1) I routinely follow-up on test results that are pending at the time of discharge as a part of patient care.

   Strongly Agree, Agree, Neutral, Disagree, Strongly Disagree

2) I use remote access during my time off to manage test results for discharged patients.

   Strongly Agree, Agree, Neutral, Disagree, Strongly Disagree, N/A

<u>Patient/Provider Notification:</u>

3) It is clear who is responsible for following up on a discharged patient's test results.

   always, often, sometimes, rarely, never

4) I consistently ensure the patient/caregiver or the primary care provider are notified of abnormal tests that result after hospital discharge.

Strongly Agree, Agree, Neutral, Disagree, Strongly Disagree

5) I have the help needed for notifying patient/caregiver or primary care provider of tests that result after discharge.

   Strongly Agree, Agree, Neutral, Disagree, Strongly Disagree

Use of IT/Tools

6) I have remote access to the EMR (ie citrix).

   Yes, no, unsure

7) I have the mobile app (ie Haiku) on my personal cell phone.

   Yes, no, unsure

8) I use manual cognitive reminders to keep track of tests pending at discharge (select all that apply):
   a) Add the patient to a reminder list (with personal notification) within the EMR
   b) Add the patient to a personal lab follow-up list in the EMR
   c) Personal electronic or handwritten notes/to-do list outside of the EMR
   d) Other: (free text)
   e) *None of the above, I do not routinely follow up on tests pending at discharge*

9) I use the patient portal (ie MyChart) to communicate test results to patients/families after hospital discharge.

   Yes, no, unsure

10) I encourage patients/families to obtain access to the patient portal (ie MyChart) to see results for tests that are pending at discharge.

   always, often, sometimes, rarely, never

Attitude about IT (Information Technology) solutions

11) I perceive management of pending tests at hospital discharge a safety issue.

   Yes, no, unsure

12) I would like our program to have a standard process for result follow-up after discharge.

   Yes, no, unsure

13) I would like that standard process to include shared responsibility with attending physicians assigned to specific day-shift providers.

Yes, no, unsure

Responder Characteristics (Demographic Data)

- My current level of training:

  PGY1, PGY2, PGY3

- I have received training/education on how to manage results follow up for tests pending at time of hospital discharge.

  Yes, no, unsure

- I would like training/education on how to manage results for tests pending at time of hospital discharge.

  Yes, no, unsure
