# Supplementary material for: Variation in the Management of Test Results after Hospital Discharge: A Pediatric Safety Concern
Source: Pediatr Qual Saf. 2026 Feb 23;11(1):e871. doi: 10.1097/pq9.0000000000000871 (PMC12928903; doi:10.1097/pq9.0000000000000871)
Supplement: Supplementary file 2 [file pqs-11-e871-s002.pdf]

Supplemental Table 1: Responder Demographics and Current Practices

**Demographics**	1-5 yrs		6-10 yrs		>10 yrs		
Number of years out of residency	6	24%	8	32%	11	44%	
Number of years you have used an EMR	3	12%	9	36%	13	52%	
	Yes		No		Unsure		
Following up on TPAD was part of my training on hospital medicine rotations during residency/fellowship	14	56%	8	32%	3	12%	
**Current Practices**	Strongly Agree / Agree		Neutral		Disagree / Strongly Disagree		p-values (agree vs neutral/ disagree)
I acknowledge all high priority alert notifications	18	86%	1	4%	2	10%	
I follow up on all high priority alert notifications received	19	90%	0	0%	2	10%	
I acknowledge all alert notifications received regardless of priority	11	52%	6	29%	4	19%	
I consistently notify my patients of abnormal test results	12	48%	8	32%	5	20%	***0.0008***
Residents	1	4%	6	24%	18	72%	
In the past year, I missed test results that were pending post-discharge	11	44%	11	44%	3	12%	
In the past year, I missed results from TPAD that led to delayed patient care	2	18%	4	36%	5	46%	
I use remote access during time off to manage alerts related to TPAD	15	60%	2	8%	8	32%	***0.0002***
Residents	2	8%	4	16%	19	76%	
I routinely follow-up on TPAD as a part of patient care (*Residents Only)*	5	20%	4	16%	16	64%	
	Always / Often		Sometimes		Rarely / Never		*p-values (always/often vs all others)*
It is clear who is responsible for following up on TPAD	3	12%	16	64%	6	24%	*0.99*
Residents	2	8%	10	40%	13	52%	
I assign a surrogate to monitor my In-Basket results when I am out of the office for an extended period (ie vacation)	1	4%	0	0%	24	96%	
I encourage patients/families to obtain access to the patient portal to follow up TPAD	9	36%	8	32%	8	32%	*0.39*
Residents	13	52%	8	32%	4	16%	

Note: All responses are from Hospitalists, unless otherwise stated (N= 25 hospitalists/fellows, N = 25 Residents). Statistical tests used to compare Hospitalists and Residents is Fisher's Exact Test. Bold = statistically significant at p-values <.05. Not all respondents answered all questions.
