# Supplementary material for: Variation in the Management of Test Results after Hospital Discharge: A Pediatric Safety Concern
Source: Pediatr Qual Saf. 2026 Feb 23;11(1):e871. doi: 10.1097/pq9.0000000000000871 (PMC12928903; doi:10.1097/pq9.0000000000000871)
Supplement: Supplementary file 3 [file pqs-11-e871-s003.pdf]

Supplemental Table 2: Responder Attitudes, Perceptions, and Preferences

**Attitudes, Perceptions, & Preferences**	Yes		No		Unsure		*p-values (yes vs no/unsure)*
I would like to receive feedback about my performance related to follow-up of high priority alert notification	10	40%	8	32%	7	28%	
I perceive the management of TPAD a safety issue in need of a system fix	20	80%	3	12%	2	8%	*0.99*
Residents	20	80%	1	4%	4	16%	
I would like to have a standard process for TPAD management	22	88%	0	0%	3	12%	*0.61*
Residents	24	96%	1	4%	0	0%	
I would like the process to include shared responsibility assigned to day-shift providers	8	32%	6	24%	11	44%	***0.023***
Residents	17	68%	2	8%	6	24%	
I received training/education on TPAD management	14	56%	8	32%	3	12%	***0.0001***
Residents	1	4%	21	84%	3	12%	
I would like training/education on TPAD management (*Residents only*)	19	76%	4	16%	2	8%	
	Strongly Agree / Agree		Neutral		Disagree / Strongly Disagree		*p-values (agree vs neutral/ disagree)*
I have the help I need to notify patients or providers of test results	3	12%	3	12%	19	76%	*0.7*
Residents	5	20%	5	20%	15	60%	
The design and function of the EMR alert notification system in contributes to providers missing test results	12	48%	11	44%	2	8%	
I receive too many alerts to easily focus on the most important ones	19	76%	1	4%	5	20%	

Note: All responses are from Hospitalists, unless otherwise stated. Statistical tests used to compare Hospitalists and Residents is Fisher's Exact Test. Bold = statistically significant at p-values <.05.
